# Exploring the role of the key gene TNFAIP3 between periodontitis and influenza A through bioinformatic analysis and molecular docking

**DOI:** 10.1371/journal.pone.0340882

**Published:** 2026-01-27

**Authors:** Mingxiang Xu, Jiaxin Shi, Yang Wang, Jie Wu, Qiaozhi Yang, Weiyun Chen, Yinpeng Li, Luwei Zhang, Xin Liu, Fu Ren, Yudong Wu, Lin Ye, Xin Li, Kebin Xu

**Affiliations:** 1 School of Medical Information Engineering, Shenyang Medical College, Shenyang, People’s Republic of China; 2 School of Public Health, Shenyang Medical College, Shenyang, People’s Republic of China; 3 Shenyang Key Laboratory of Prevention and Treatment of Systemic Important Diseases Associated with Oral Diseases, Shenyang, People’s Republic of China; 4 Affiliated 242 hospital, Shenyang Medical College, Shenyang, People’s Republic of China; 5 School of Stomatology, Shenyang Medical College, Shenyang, People’s Republic of China; 6 Liaoning Province Key Laboratory for Phenomics of Human Ethnic Specificity and Critical Illness (LPKL-PHESCI), Shenyang, People’s Republic of China; 7 Shenyang Key Laboratory for Phenomics, Shenyang, People’s Republic of China; Oklahoma Medical Research Foundation, UNITED STATES OF AMERICA

## Abstract

Recent studies have hinted at a link between periodontitis (PD) and influenza A (IA). Therefore, this study aims to identify key genes common to both diseases by studying *Porphyromonas gingivalis* (*P.gingivalis*) and the H1N1 virus, the main pathogen of PD and IA, evaluating the promise of these genes as biomarkers, and providing future therapeutic strategies for both diseases. Our research employs a wide range of methods, including bioinformatics analysis, drug prediction and molecular docking. Ultimately, machine learning algorithms identified TNFAIP3 as a common key gene for PD and IA, and further identified five drugs: Vemurafenib, Metformin, Dexamethasone, Tretinoin and Imatinib with potential for combined treatment of PD and IA. Our study reveals the shared mechanism and immune profile of TNFAIP3 as a key gene in PD and IA, which lays a theoretical foundation for future targeted therapies based on the shared mechanism of the two diseases.

## Introduction

Periodontitis (PD) is a complex disease whose main cause is plaque [1]. This disease induces an inflammatory reaction in the organism, damaging the supporting tissues of the teeth [2,3], and in severe cases, it can lead to tooth loss [4]. *Porphyromonas gingivalis* (*P.gingivalis*) is a gram-negative anaerobic bacterium that is an important pathogen causing PD [5]. The mechanisms of interaction between PD and systemic diseases have not been well studied. But research suggests that periodontal pathogens and the immune responses they elicit are implicated in the pathogenesis of a variety of systemic diseases, such as diabetes mellitus, cardiovascular disease, Alzheimer’s disease, chronic obstructive pulmonary disease, chronic kidney disease, and cancer [6–8]. The present findings indicate that infection of the host by *P.gingivalis* is mainly dependent on flagella [9]. After invading the host’s tissues, *P.gingivalis* secretes gingival proteases to obtain nutrients and disrupt the host’s immune response [10–12].

H1N1 is a pathogen of influenza, which is a subtype of influenza A (IA) virus. Since the 2009 pandemic, H1N1 influenza has caused varying degrees of disease burden globally every year [13]. Studies have shown that the pathogenesis of both *P.gingivalis* infections and H1N1 infections involves the PI3K/AKT/mTOR signaling pathway [14], and we believe that there are additional links between them. Therefore, in this study, we used bioinformatics and machine learning methods to investigate the association between *P.gingivalis* infection and H1N1 infection. Bioinformatics machine learning and deep learning help to comprehensively analyze diseases at the genetic level as a way to identify biomarkers and therapeutic targets for disease diagnosis [15]. Through bioinformatics and machine learning analyses, we identified common differential genes and molecular mechanisms between *P.gingivalis* infections and H1N1 infections, on the basis of which we have found promising drugs for the combined treatment of PD and IA, providing new insights into the study of the two diseases.

## Materials and methods

### Data collection

We downloaded the *P.gingivalis* infection related datasets GSE12121 (con = 4, *P.gingivalis* = 4) [16], GSE9723 (con = 4, *P.gingivalis* = 4) [17] and GSE16134 (con = 69, *P.gingivalis* = 241) [18,19], and the H1N1 virus infection related datasets GSE36553 (con = 5, H1N1=6) [20], GSE48466 (con = 3, H1N1=6) [21] and GSE106279 (con = 6, H1N1=6) [22] from the Gene Expression Omnibus (GEO) (https://www.ncbi.nlm.nih.gov/geo/) database. We obtained 4 groups of mock-infected HIGK cells as control and 4 groups of *P.gingivalis*-infected HIGK cells as experimental groups from GSE12121 (GPL570). We selected 4 groups of mock-infected HIGK cells and 4 groups of *P.gingivalis*-infected HIGK cells from GSE9723 (GPL96). GSE16134 (GPL570) includes 310 gingival tissue samples from 120 patients with periodontitis who underwent periodontal surgery, including 69 normal and 241 periodontitis samples. We selected 5 groups of mock-infected A549 cells and 6 groups of A549 cells 48 hours after H1N1 infection from GSE36553 (GPL6947). We selected 3 groups of uninfected wd-NHBE cells and 6 groups of pandemic H1N1 influenza virus-infected wd-NHBE cells from GSE48466 (GPL570). GSE106279 (GPL17586) includes 6 sets of mock-infected A549 cells and 6 sets of H1N1-infected A549 cells. Subsequent differential gene analysis was performed by using R software (version 4.3.3).

### Differential gene analysis

We first merged the *P.gingivalis* infection dataset (GSE12121 and GSE9723) and the H1N1 infection dataset (GSE36553 and GSE48466) separately. To ensure the quality of the data, we used the “sva” R package to normalize the two disease datasets and eliminate the batch effect [23]. Subsequently, we used the “limma” package to identify differentially expressed genes in the two datasets, with the criteria for significant differential expression being log |fold change| > 1 and adjusted P value<0.05 [24]. Finally, volcano maps and heatmaps were generated to visualize the differentially expressed genes.

### Identification and enrichment analysis of cDEGs

In order to identify common differential genes between *P.gingivalis* infection and H1N1 infection, we intersected the differential genes identified from the two datasets to create a Venn diagram. Subsequently, these cDEGs were enriched for gene ontology (GO), including biological process (BP), cellular component (CC) and molecular function (MF), as well as Kyoto Encyclopedia of Genes and Genomes (KEGG) signaling pathways using the “clusterProfiler” package [25]. This analysis is important for understanding the functional roles involved in cDEGs.

### Identification of key genes

In this study, we employ two advanced machine learning methods: the Least Absolute Shrinkage and Selection Operator (LASSO) and the Support Vector Machine (SVM). LASSO is an extension of the linear regression model that introduces a penalty term for the compression and selection of model coefficients, thus solving the covariance and overfitting problems [26]. In this article, we used 5-fold cross validation with Mean Squared Error as the evaluation metric. SVM is a widely used supervised learning algorithm that shows good performance even with small sample sizes, and is well suited to handle small and medium-sized datasets [27]. We control the number of features removed in each iteration to 8 and use 8-fold cross validation. These machine learning algorithms can help us identify the most important key genes from a large number of genes.

### Validation and enrichment analysis of key genes

We intersected the results obtained from the two machine learning methods and plotted a Venn diagram to identify the genes in the intersection set as the common key genes for the two diseases. To verify the reliability of the machine learning results, we plotted the ROC curves of the key genes using the “pROC” package [28]. Moreover, we verified the expression levels of common key differential genes in two external datasets GSE16134 and GSE106279. Finally, we explored the roles of the common key genes in the two diseases by GO enrichment and KEGG enrichment.

### Analysis of immune cell infiltration

CIBERSORT is a deconvolution technique that characterizes the expression of different cell types by isolating them from complex tissue samples and thus estimating the relative proportions of these cells in the sample [29]. In this study, we used the CIBERSORT method to assess the level of immune infiltration of 22 known immune cell subtypes in the dataset. p < 0.05 was considered statistically significant for immune cell infiltration. Subsequently, to explore immune cells associated with the key gene TNFAIP3, we performed Spearman correlation analysis.

### Drug prediction and molecular docking based on key genes

Drug Gene Budger (DGB) (https://maayanlab.cloud/DGB/) is a drug repositioning tool that allows researchers to select which genes they want to up or down regulate, and DBG is able to predict the drug that will have the greatest impact on the target gene [30]. DBG gives results from the Library of Integrated Network-Based Cellular Signatures (LINCS) L1000 dataset [31], the Connectivity Map (CMap) dataset [32] and the Gene Expression Omnibus (GEO) database [33]. In this study, we selected compounds from the GEO database that significantly down-regulated TNFAIP3 expression with p-value<0.05, q-value<0.05 and log2|FC| > 1. The structures of TNFAIP3 were obtained from Pubchem (https://pubchem.ncbi.nlm.nih.gov/) and those of small molecule compounds were obtained from DrugBank Online (https://go.drugbank.com/). We used AutoDockTools (version 1.5.7) to analyze the best docked conformations of proteins and small molecule compounds, and the final results were visualized by PyMol (version 3.0.4).

### Molecular dynamic simulation

Molecular dynamics simulations (MD) were performed using the Gromacs2022 program with GAFF force field for small molecules [34], AMBER14SB force field for proteins and TIP3P water model to merge the files of proteins and small molecule ligands to construct a simulation system for the complexes. The simulations were performed under constant temperature and pressure and periodic boundary conditions. During the MD simulations, all hydrogen bonds involved were constrained using the LINCS algorithm with an integration step of 2 fs. Electrostatic interactions were calculated using the (Particle-mesh Ewald) PME method with a cutoff value of 1.2 nm, and non-bonded interactions with a cutoff value of 10 Å, which was updated every 10 steps. The simulation temperature was controlled by the V-rescale temperature coupling method at 298 K, and the pressure was controlled by the Berendsen method at 1 bar. 100 ps of NVT and NPT equilibrium simulations were carried out at 298 K. 100 ns of MD simulations were performed for the complex system, and the conformation was saved every 10 ps. After the simulations were completed, the trajectories were analyzed using VMD and pymol. We used Root Mean Square Deviation (RMSD) and Radius of Gyration (Rg) to reflect the stability of protein binding to small molecules.

### Statistical analysis

R software (version 4.3.3) was used for statistical analysis. P < 0.05 was considered statistically significant.

## Results

### Merging of datasets

**Fig 1:**
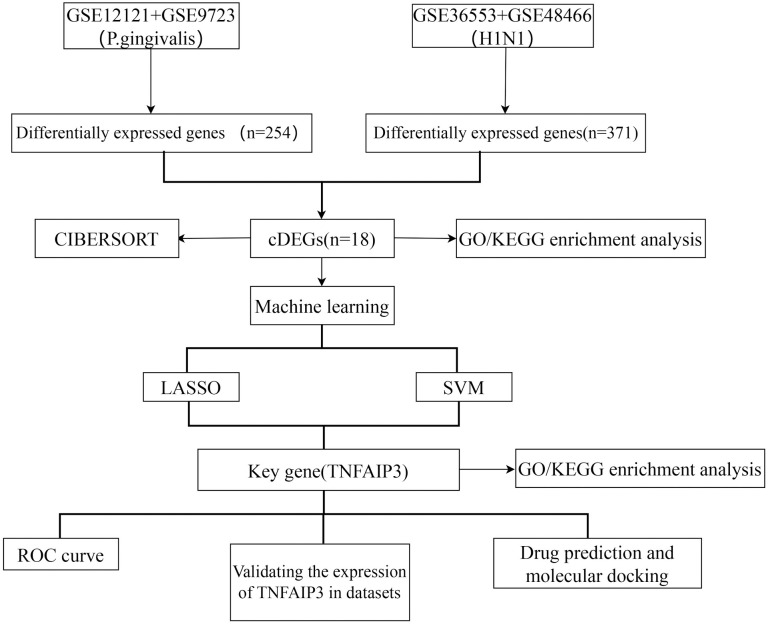
Flowchart of this study. cDEGs: common differentially expressed genes. GO: Gene Ontology. KEGG: Kyoto Encyclopedia of Genes and Genomes. LASSO: least absolute shrinkage and selection operator. SVM: support vector machine. ROC curve: receiver operating characteristic curve.

**Fig 2:**
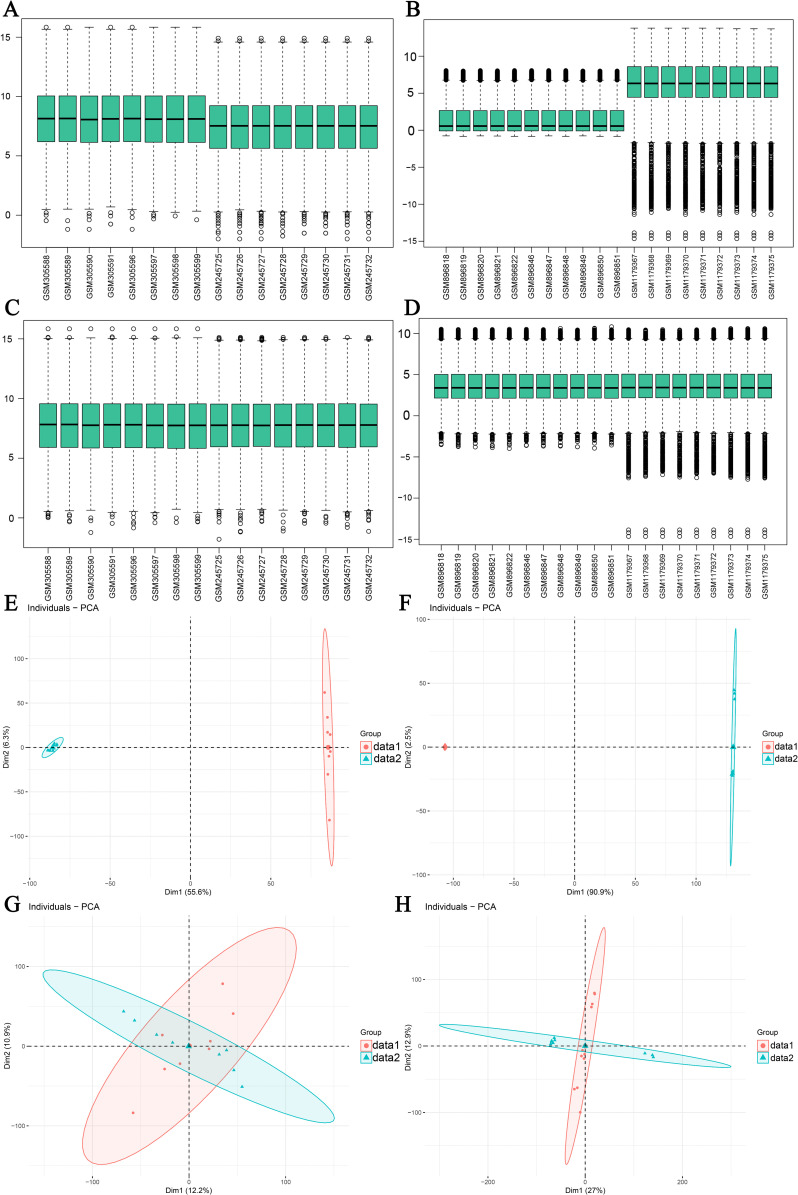
Merging of datasets. (A, B) Standardized previous *P.gingivalis* infection (A) and H1N1 infection (B) datasets. (C, D) *P.gingivalis* infection (C) and H1N1 infection (D) datasets after standardization. X: The name of the sample in the GEO dataset. Y: Values for gene expression levels. (E, F) PCA results prior to removal of batch effects in *P.gingivalis* infected (E) and H1N1 infected (F) datasets. (G, H) PCA results after removal of batch effects in *P.gingivalis* infected (G) and H1N1 infected (H) datasets. PCA: principal component analysis.

The flowchart of this study is shown in Fig 1. We merged datasets downloaded from the Gene Expression Omnibus (GEO) database. In order to ensure the quality of the data, we first normalized the merged data and used box plots to show the distribution of expression values (Fig 2A-2D). Moreover, in order to detect the removal of batch effects for data from different sources, we performed principal component analysis (PCA) on the merged dataset. The results showed that group effects across sources were largely eliminated in the merged dataset (Fig 2E-2H).

### Identification of differentially expressed genes

**Fig 3:**
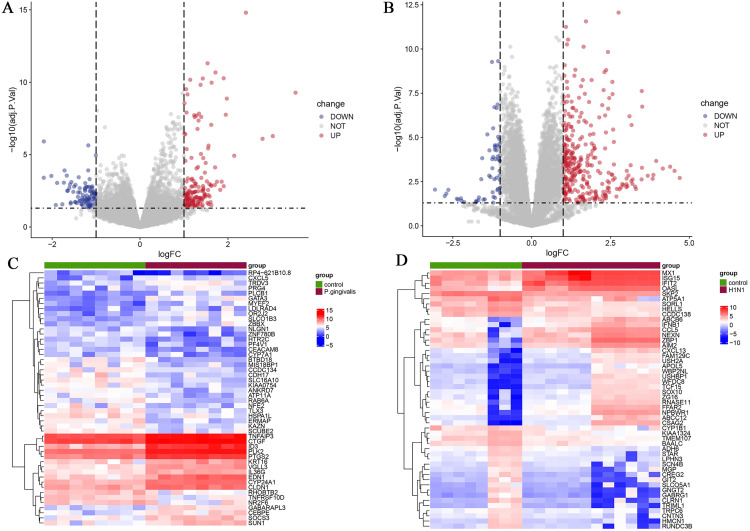
Visualization of differentially expressed genes. (A) Volcano plot showing the differentially expressed genes (DEGs) of *P.gingivalis* infection. (B) Volcano plot showing the differentially expressed genes (DEGs) of H1N1 infection. (C) Heatmap of DEGs in *P.gingivalis* infection. (D) Heatmap of DEGs in H1N1 infection.

We performed differential gene expression analysis on the two merged datasets and identified 254 differentially expressed genes (DEGs) in *P.gingivalis* infection and 371 DEGs in H1N1 infection. There were 90 genes down-regulated and 164 genes up-regulated in the *P.gingivalis*-infected group. In the H1N1-infected group, 60 genes were downregulated and 311 genes were upregulated. We used volcano plots to visualize DEGs, highlighting down and up regulated genes (Fig 3A and 3B). In addition, we used heatmaps to show the expression patterns and clustering of DEGs in gene expression profiles (Fig 3C and 3D).

### Enrichment analysis of common differential genes

**Fig 4:**
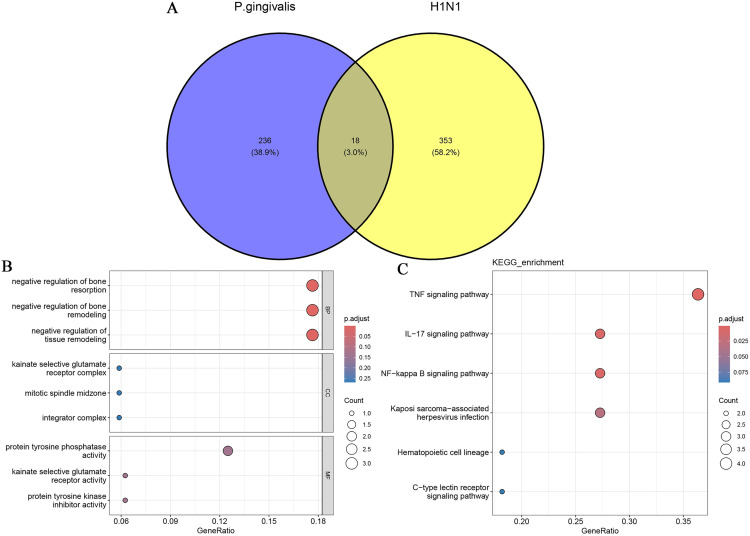
Enrichment analysis of cDEGs. (A) Venn diagram of overlapping genes. (B) Bubble diagram showing the results of GO enrichment analysis of overlapping genes. (C) Bubble diagram showing the results of KEGG enrichment analysis of overlapping genes.

To identify common differential genes (cDEGs) for *P.gingivalis* infection and H1N1 infection, we intersected the results of the differential gene expression analyses of the two diseases and obtained 18 overlapping genes (Fig 4A). Enrichment analysis of these overlapping genes showed that in the GO enrichment analysis (Fig 4B), biological processes (BP) are mainly enriched for negative regulation of bone resorption, negative regulation of bone remodeling and negative regulation of tissue remodeling. Cellular Components (CC) are mainly enriched for kainate selective glutamate receptor complex, mitotic spindle midzone and integrator complex. Molecular functions (MF) are mainly enriched for protein tyrosine phosphatase activity, kainate selective glutamate receptor activity and protein tyrosine kinase inhibitor activity. The results of KEGG analysis were mainly enriched for TNF signaling pathway, IL-17 signaling pathway, NF-kappa B signaling pathway, Kaposi sarcoma-associated herpesvirus infection, Hematopoietic cell lineage, and C-type lectin receptor signaling pathway (Fig 4C). The results of these enrichment analyses emphasize the important role of inflammation-related mechanisms in both diseases.

### Machine learning identified key genes

**Fig 5:**
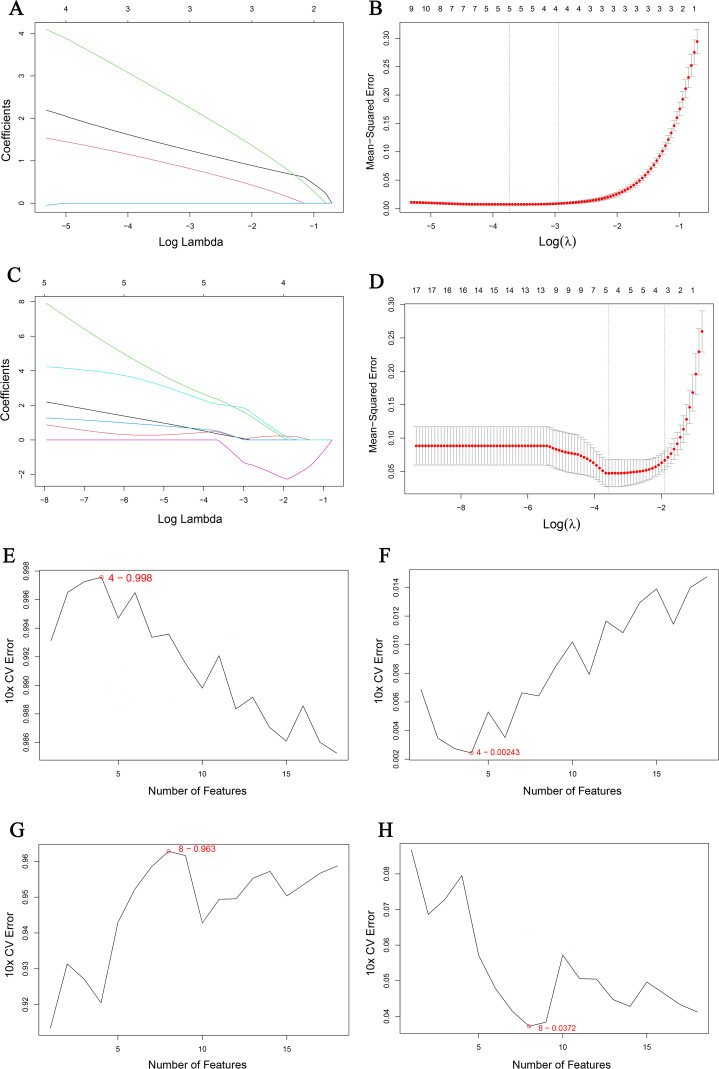
Results of machine learning. (A, B) LASSO regression of the *P.gingivalis* infection dataset. (C, D) LASSO regression of the H1N1 infection dataset. (E, F) Number of genes in the *P.gingivalis* infection dataset for which the SVM algorithm has the highest accuracy rate and the lowest error rate. (G, H) Number of genes in the H1N1 infection dataset for which the SVM algorithm has the highest accuracy rate and the lowest error rate.

To further identify key genes from the 18 overlapping genes, we used two machine learning methods: LASSO regression and SVM. The LASSO regression identified three key genes in the *P.gingivalis* infection dataset (Fig 5A and 5B) and six key genes in the H1N1 infection dataset (Fig 5C and 5D). We visualized the correct and error rates of the SVM algorithm. The results showed that in the *P.gingivalis* infection dataset, the SVM model had the highest accuracy when four genes were included in the analysis (Fig 5E and 5F). In the H1N1 infection dataset, the SVM model had the highest accuracy when eight genes were included in the analysis (Fig 5G and 5H).

### Validation and enrichment analysis of common key genes

**Fig 6:**
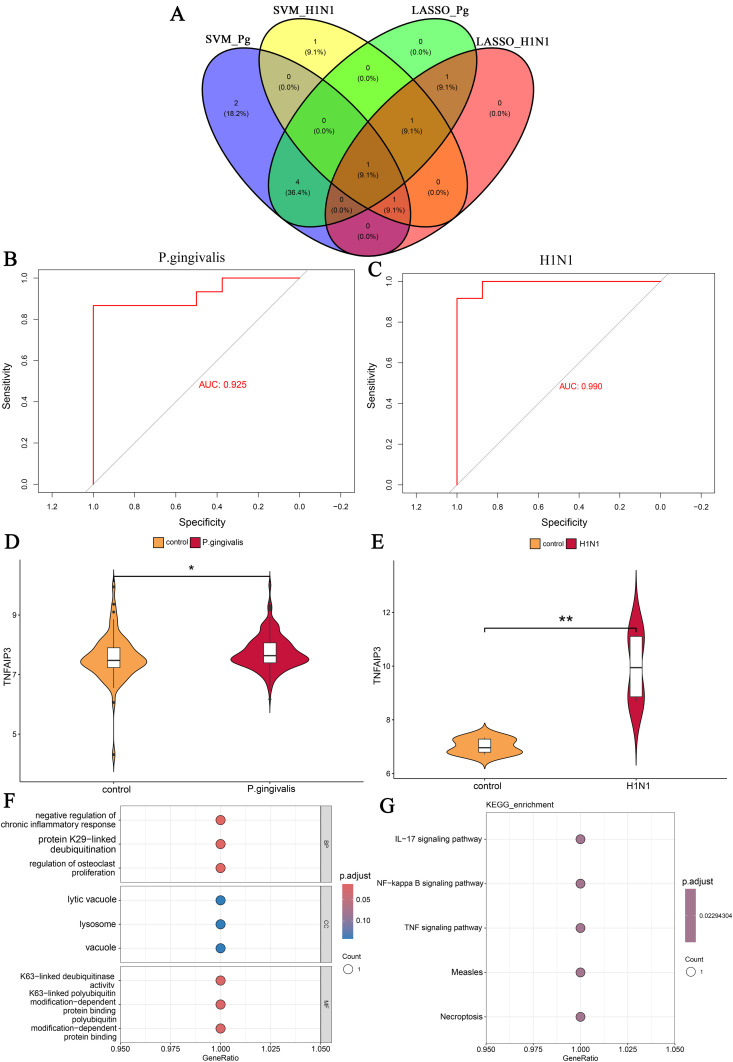
Results of validation and enrichment analysis of common key genes. (A) Venn diagram of machine learning results. (B) ROC curve of TNFAIP3 in the *P.gingivalis* infection dataset (AUC = 0.925). (C) ROC curve of TNFAIP3 in the H1N1 infection dataset (AUC = 0.990). (D) Expression of TNFAIP3 in GSE16134. (E) Expression of TNFAIP3 in GSE106279. (F) GO enrichment analysis of TNFAIP3. (G) KEGG enrichment analysis of TNFAIP3. * and ** indicate significant difference from the control group. * P < 0.05, ** P < 0.01.

We used a Venn diagram to visualize the overlapping portion of the results from the two machine learning methods (Fig 6A). The gene in the overlapping part is Tumor Necrosis Factor Alpha-Induced Protein 3 (TNFAIP3). To validate the accuracy of the machine learning algorithm, we plotted the ROC curves of the TNFAIP3 gene in both the *P.gingivalis* infection dataset and the H1N1 infection dataset (Fig 6B and 6C). The results showed that the results of the machine learning method had high diagnostic value in both datasets. Moreover, we validated the expression levels of TNFAIP3 in both diseases in two external datasets, GSE16134 and GSE106279. The expression of TNFAIP3 was significantly upregulated in both diseases compared to controls (Fig 6D and 6E). Subsequently, we performed GO enrichment analysis and KEGG enrichment analysis of the TNFAIP3 gene. In GO enrichment (Fig 6F), BP are mainly enriched for negative regulation of chronic inflammatory response, protein K29-linked deubiquitination and regulation of osteoclast proliferation. CC are mainly enriched for lytic vacuole, lysosome and vacuole. MF are mainly enriched for K63-linked deubiquitinase activity, K63- linked polyubiquitin modification-dependent protein binding and polyubiquitin modification-dependent protein binding. It is important to note that only one gene was included in our GO analysis, which may have implications for the significance of the results and the integrity of the biological pathway. For example, CC was not statistically significant in our GO analysis results. KEGG are mainly enriched for IL-17 signaling pathway, NF-kappa B signaling pathway, TNF signaling pathway, Measles and Necroptosis (Fig 6G). These results reveal that TNFAIP3 plays an important role as a common key gene in the development of both diseases.

### Analysis of immune cell infiltration

**Fig 7:**
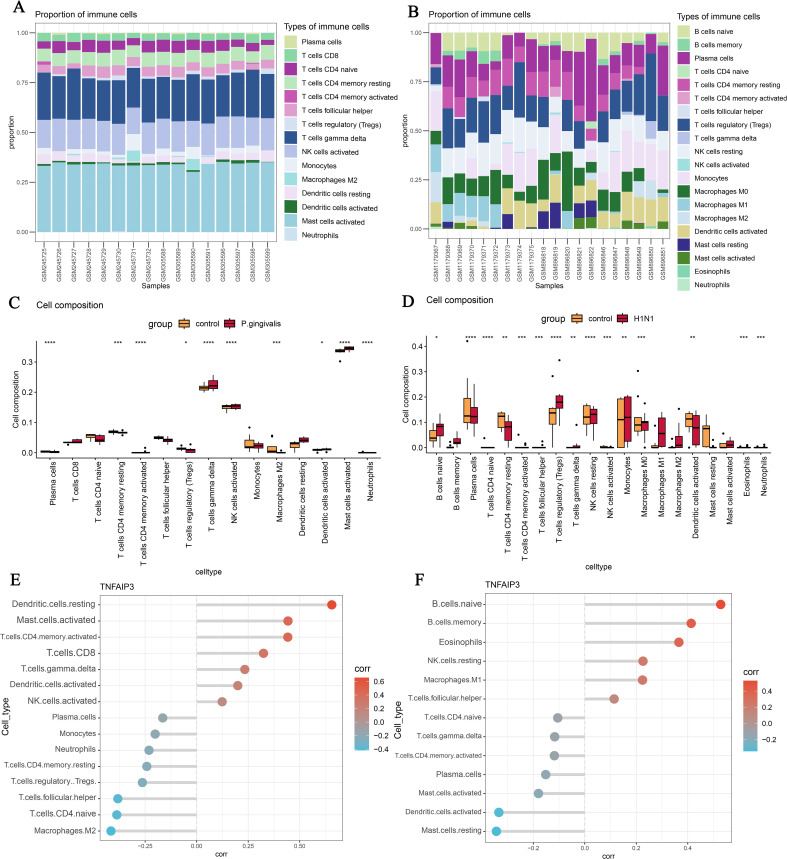
Analysis of immune cell infiltration. (A) Bar graph of immune cell infiltration in the *P.gingivalis* infection dataset. (B) Bar graph of immune cell infiltration in the H1N1 infection dataset. (C) Comparison of differences in immune cell abundance between *P.gingivalis*-infected and control groups. (D) Comparison of differences in immune cell abundance between H1N1-infected and control groups. (E) Correlation of TNFAIP3 with immune cells in the *P.gingivalis* infection dataset. (F) Correlation of TNFAIP3 with immune cells in the H1N1 infection dataset. *, **, *** and **** indicate significant difference from the control group. * P < 0.05, ** P < 0.01, *** P < 0.001, **** P < 0.0001.

**Fig 8:**
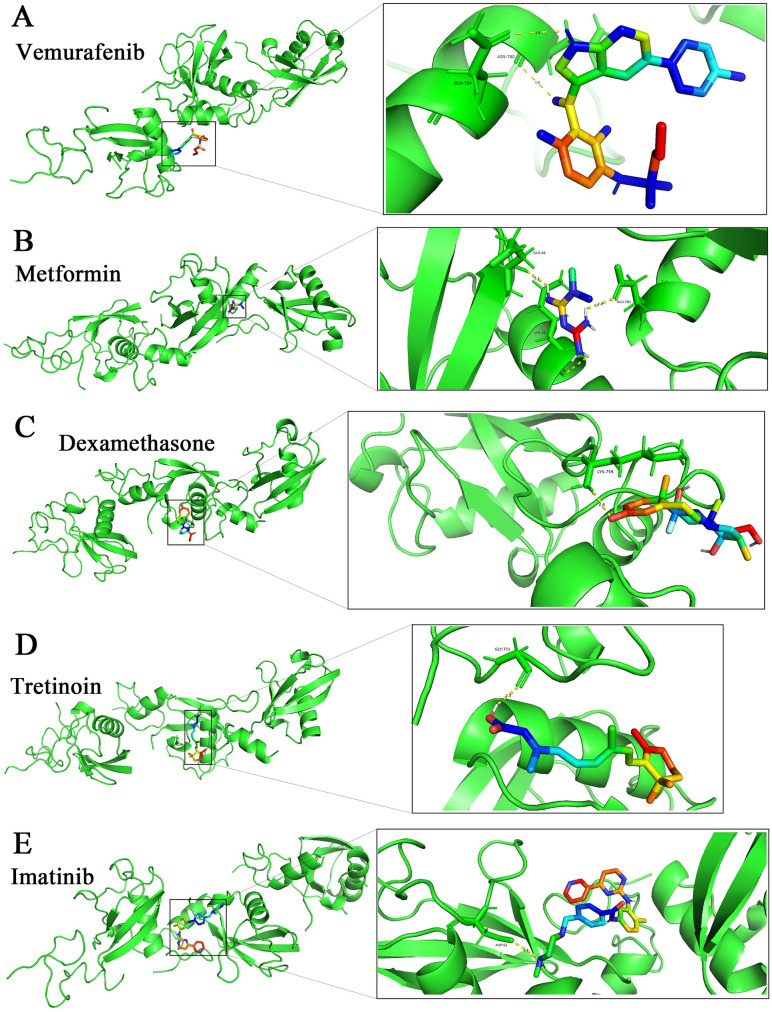
Optimal conformations of five drug molecules of molecular docking with TNFAIP3 protein. (A) Optimal conformation of Vemurafenib with TNFAIP3. (B) Optimal conformation of Metformin with TNFAIP3. (C) Optimal conformation of Dexamethasone with TNFAIP3. (D) Optimal conformation of Tretinoin with TNFAIP3. (E) Optimal conformation of Imatinib with TNFAIP3.

We used the CIBERSORT algorithm to analyze the correlation of gene expression profiles with 22 known immune cells in both disease datasets. Bar graphs were used to visualize the results of immune infiltration (Fig 7A and 7B). The findings showed that in *P.gingivalis* infection, Plasma cells, T cell subsets, NK cells, Mast cells and Neutrophils were the major immune cell infiltrating subpopulations (Fig 7C). In H1N1 infection, Plasma cells, T cell subsets, NK cells, Monocytes, Eosinophils and Neutrophils were the major immune cell infiltrating subpopulations (Fig 8D). Moreover, we analyzed the correlation of the key gene TNFAIP3 with immune cells in both diseases. The results showed that in *P.gingivalis* infection, TNFAIP3 was significantly positively correlated with resting-state dendritic cells and significantly negatively correlated with Macrophages M2, and also correlated more significantly with various T-cell subpopulations (Fig 8E). In H1N1 infection, TNFAIP3 was significantly positively correlated with B-cell subsets and significantly negatively correlated with mast cells in the resting state and dendritic cells in the activated state (Fig 8F). These results showed us the infiltration status of immune cells in both diseases.

### Drug prediction and molecular docking based on key gene

In this study, we explored molecules that downregulate TNFAIP3 expression using Drug Gene Budger (DGB). Five drug molecules were screened by using p-value<0.05, q-value<0.05 and log2 |fold change| < 1: Vemurafenib, Metformin, Dexamethasone, Tretinoin and Imatinib (S1 Table). Subsequently, we used molecular docking to predict the optimal conformations of the five drug molecules bound to TNFAIP3. Fig 8A-8E show us in detail the specifics of the optimal conformations of the five drug molecules with TNFAIP3. S2 Table shows us the binding energies, the number of hydrogen bonds formed and the locations of the binding sites of the TNFAIP3 proteins with the five drug molecules. The results show that all five drug molecules have low binding energies and form stable structures at their binding sites with the target protein, and Imatinib showed the highest docking score with TNFAIP3 at −8.1 kcal/mol. Therefore, the five drug molecules predicted in this study may have the potential to treat influenza A patients suffering from periodontitis by down-regulating the expression of TNFAIP3.

### Molecular dynamic simulation

**Fig 9:**
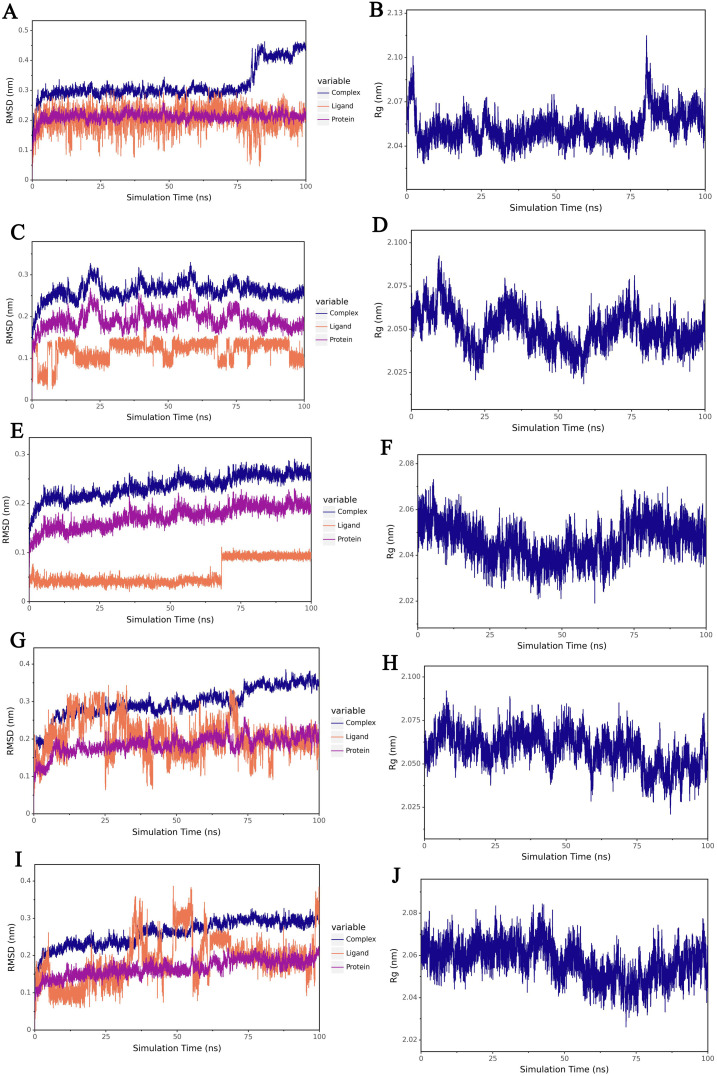
Molecular dynamics simulation results of TNFAIP3 binding to five drug molecules. (A) RMSD of TNFAIP3-Vemurafenib. (B) Rg of TNFAIP3-Vemurafenib. (C) RMSD of TNFAIP3-Metformin. (D) Rg of TNFAIP3-Metformin. (E) RMSD of TNFAIP3-Dexamethasone. (F) Rg of TNFAIP3-Dexamethasone. (G) RMSD of TNFAIP3-Tretinoin. (H) Rg of TNFAIP3-Tretinoin. (I) RMSD of TNFAIP3-Imatinib. (J) Rg of TNFAIP3-Imatinib. RMSD: root mean square deviation. Rg: radius of gyration.

RMSD is a common indicator of the difference between two structures, which reflects the similarity of two molecules’ conformations by comparing the spatial coordinates of the corresponding atoms in the two molecules, and is used to track the changes of the molecular structure during the simulation process with respect to the initial structure, and to observe whether the change amplitude tends to be stabilized. The lower the RMSD, the closer the two structures are. As can be seen in Fig 9A, C, E, G and I, the RMSDs of the structures of the complexes of TNFAIP3 with the five drug molecules were gradually stabilized with the simulation, indicating that the structures of the complexes were gradually stabilized. Rg is the root-mean-square distance of all atoms in a molecule relative to the center of mass, reflecting the distribution range of molecular atoms relative to the center of mass, and is used as an important parameter to measure the degree of compactness of the overall structure of protein-small molecules. The smaller the radius of gyration Rg is, the more compact the molecule is; the larger the radius of gyration is, the looser the molecule is, and it may be in a fluffy state. As can be seen from Fig 1B, D, F, H and I, the Rg of the complexes gradually stabilizes with the simulation, which indicates the gradual stabilization of the complex structure. All these results indicate that TNFAIP3 can bind to five drug molecules to form a stable structure.

## Discussion

Periodontitis (PD) and influenza A (IA) are two very common diseases whose pathophysiologic mechanisms are unknown. In order to identify the pathophysiologic mechanisms that are commonly associated with both, we focused on *P.gingivalis*, the major pathogen of PD, and the H1N1 virus, the major pathogen of IA. In our differential gene analysis of the two diseases, we identified 254 DEGs and 371 DEGs, and identified 18 overlapping genes from these two differential gene sets. Enrichment analysis of 18 overlapping genes demonstrated biological processes (BP), cellular composition (CC), molecular functions (MF) and signaling pathways that may be associated with both diseases. To further identify the key genes among these overlapping genes, we used two advanced machine learning algorithms: LASSO and SVM. By combining the results of both approaches, we identified the key gene TNFAIP3. In both diseases, TNFAIP3 is an upregulated gene. Subsequently, we analyzed the immunological profiles of the DEGs in the two diseases by using the CIBERSORT algorithm and Spearman’s correlation analysis, highlighting the role of key genes in immune regulation. By using TNFAIP3 as a target protein, we predicted five promising drugs for the simultaneous treatment of PD and IA. In summary, these findings suggest that TNFAIP3 may be a key factor in the development and progression of PD and IA, emphasizing their potential as diagnostic markers and therapeutic targets.

The proteins encoded by TNFAIP3 are zinc finger proteins and ubiquitin editing enzymes. Among them, ubiquitin editing enzymes have ubiquitin ligase and deubiquitinase activities and are involved in cytokine-mediated inflammatory and immune responses [35]. In the NF-κB signaling pathway, TNFAIP3 negatively regulates NF-κB activation through its deubiquitinating enzyme activity. When Toll-like receptor (TLR) or tumor necrosis factor receptor (TNFR) is activated, TNFAIP3 terminates NF-κB signaling by removing the ubiquitin chain of key signaling molecules (e.g., RIP1, TRAF6), preventing excessive inflammatory responses [36,37]. Periodontitis is a chronic inflammatory disease triggered by plaque microorganisms in which bacterial components (e.g., lipopolysaccharides) activate the IκB kinase (IKK) complex by binding to host cell-surface receptors (e.g., TLR2/TLR4), leading to the phosphorylation and degradation of IκB, the release of NF-κB into the nucleus, and the initiation of proinflammatory factors (e.g., TNF-α, IL-1β, and IL-6) and matrix metallo proteases (MMPs) transcription [38]. Sustained NF-κB activation leads to periodontal tissue inflammation, osteoclast activation, and connective tissue degradation. Furthermore, NF-κB promotes RANKL expression and exacerbates alveolar bone destruction [39]. In H1N1 influenza, viral RNA is recognized by host intracellular receptors (e.g., RIG-I or TLR7), which activate the IKK complex and release NF-κB [40]. Viral proteins can also directly inhibit IκB and enhance NF-κB activity. NF-κB drives a storm of cytokines (e.g., IL-6, IL-8, and TNF-α), leading to alveolar epithelial damage, pulmonary edema, and multi-organ failure [41,42]. Excessive inflammation is the leading cause of death from severe H1N1 infection. TNFAIP3 attenuates inflammatory injury by inhibiting the NF-κB pathway in both PD and IA diseases, but its regulation may be tissue-specific. For example, it primarily targets TLR signaling in periodontitis, whereas it acts more on the RIG-I pathway in influenza [43]. IL-17 promotes the NF-κB and MAPK pathways through activation of the TAK1 kinase, which induces the production of inflammatory factors. TNFAIP3 restricts IL-17-mediated inflammatory responses by inhibiting ubiquitination of TAK1 and blocking downstream signaling [44]. In periodontitis, IL-17 exacerbates periodontal tissue destruction by promoting neutrophil recruitment, proinflammatory factor release, and osteoclast differentiation [45]. In a TNFAIP3-deficient mouse model of periodontitis, IL-17 levels were significantly elevated, accompanied by more severe bone resorption and inflammatory responses, and the primary mechanism is that TNFAIP3 reduces osteoclast differentiation by inhibiting TRAF6 ubiquitination downstream of IL-17R signaling [46,47]. IL-17 aggravates lung tissue injury caused by H1N1 virus infection by recruiting neutrophils and activating macrophages [48]. TNFAIP3 can reduce the release of pro-inflammatory factors (e.g., IL-6, IL-1β) by inhibiting the TLR/IL-17R signaling pathway, thereby alleviating lung injury [49]. There are no direct studies examining the interaction between periodontitis and H1N1 influenza via the IL-17 pathway, but both involve IL-17-mediated hyperinflammatory responses, and oral infections (e.g., periodontitis) may exacerbate respiratory viral infection outcomes through systemic inflammation. In periodontitis, TNF-α is a central factor mediating periodontal tissue destruction, which promotes osteoclast differentiation and leads to alveolar bone resorption. TNFAIP3 may indirectly reduce RANKL-mediated bone destruction by inhibiting the TNF signaling pathway [50]. TNF-α has a dual role in the excessive immune response triggered by H1N1 influenza virus infection. TNF-α inhibits viral replication and facilitates viral clearance by activating macrophages and CD8 + T cells [51]. However, excessive TNF-α synergizes with IL-6 and IL-1β, leading to increased vascular permeability, pulmonary edema, and multi-organ damage, especially in acute respiratory distress syndrome (ARDS) [52]. TNFAIP3 terminates multiple inflammatory signals (e.g., TLR, NLR pathways) through ubiquitin editing enzyme activity, targeting TNF receptor-associated factor (TRAF) and receptor-interacting proteins (RIPs), a mechanism that is universal in bacterial inflammation (periodontitis) and viral infections (H1N1).

Immune cell infiltration analysis revealed the role of immune cells in PD and IA. Our results showed that plasma cells, NK cells and Neutrophils infiltrated at significantly higher levels in PD combined with IA. Plasma cells are the main source of the anti-inflammatory factors IL-35 and IL-37 in the gingival tissues of patients with periodontitis [53]. A study using a mouse model of periodontitis found that NK cells produce pro-inflammatory cytokines through direct interaction with periodontal pathogens, which in turn may lead to bacterial alveolar bone loss [54]. Previous studies have suggested that different NK cell subsets may correlate with the severity of influenza [55]. In patients with severe H1N1 influenza, the proportion of NKp44 + cells was significantly increased, while the proportion of NKp44- cells was significantly decreased [56]. Increased numbers of neutrophils in periodontal tissues caused by infection with periodontal pathogens including *P.gingivalis* is one of the hallmarks of PD [57]. Increased numbers of neutrophils in the lungs of patients with IA are usually caused by elevated levels of inflammatory cytokines [58], and these enriched neutrophils are able to prevent influenza-induced lung damage [59]. It is notable that the key gene TNFAIP3, which we identified by using a machine learning algorithm, did not function on the same immune cells in both diseases. For PD, given its chronic inflammatory nature and the well-established anti-inflammatory effects of TNFAIP3, directly upregulating TNFAIP3 expression or enhancing its function may serve as an effective strategy to target immune cells and suppress immune inflammatory responses. However, when patients also have IA, simply enhancing or suppressing TNFAIP3 may not be advisable. This is because TNFAIP3 plays a dual role: it can suppress antiviral immune responses to promote viral replication, yet it also functions as a host protective factor to prevent immunopathology. Therefore, given TNFAIP3’s distinct role in PD and IA interactions with immune cells, intervention targeting TNFAIP3 may require precisely balancing immunosuppression and antiviral response, such as by targeting specific ubiquitination sites or downstream pathways.

We screened five potential drug molecules that may modulate TNFAIP3 expression to affect PD with IA: Vemurafenib, Metformin, Dexamethasone, Tretinoin and Imatinib. The results of molecular docking experiments indicate that all five drug molecules can act on the TNFAIP3 protein and form a stable structure. Vemurafenib is a selective BRAF inhibitor that prevents the growth of cancer cells by inhibiting the activity of BRAF mutant proteins, and is approved for the treatment of unresectable or metastatic melanoma bearing the V600E mutation of BRAF [60]. Metformin is mainly used for the treatment of type II diabetes mellitus, especially in patients who are overweight or obese [61]. In recent years, research on Metformin has focused on its anti-aging and anticancer effects [62,63]. Dexamethasone can inhibit the production and release of inflammatory mediators by binding to intracellular glucocorticoid receptors and affecting gene transcription, thereby reducing inflammatory responses and suppressing immune system overreactions [64]. Tretinoin is a vitamin A derivative that is commonly used in the treatment of skin disorders, especially acne and skin aging [65]. It has been suggested that retinoids, including Tretinoin, may play a role in the regeneration of periodontal tissues [66,67]. Imatinib, a tyrosine kinase inhibitor, is used in the targeted treatment of chronic myeloid leukemia and gastrointestinal mesenchymal tumors [68]. These results provide a research direction for targeting TNFAIP3 for the treatment of PD with IA, although further validation through in vivo and in vitro experiments is needed.

In this study, we integrated a variety of advanced techniques including bioinformatics, machine learning, drug prediction, and molecular docking as a way to identify the key gene TNFAIP3 and delve deeper into its role in the immune response. The reliability of our findings was enhanced by validation in the dataset. However, our study does have some limitations. Firstly, the sample size of the dataset we included is relatively small, which may introduce some bias in the analysis results. To address this issue, we will use a dataset with a larger sample size for future analysis. Secondly, the samples we used for bioinformatics analysis were from different tissues, which had some impact on the final results. For example, when performing gene ontology expression analyses of cDEGs, the biological functions of different tissues will differ. Thirdly, we recognize the need for further experimental validation of the role of key genes in signaling pathways. Considering the existence of tissue-specific effects, an ideal therapeutic strategy should integrate high-precision diagnostic technologies to identify the expression status of TNFAIP3 in diseased tissues. Based on its expression levels across different tissues and its distinct functions in specific cell types, personalized interventions should be implemented using precise, spatially controllable delivery platforms. Future research will focus on developing precise modulators targeting TNFAIP3 to optimize its immunoregulatory effects across diverse diseases and tissues. Alternatively, nanotechnology may be employed to achieve targeted delivery of TNFAIP3 or its modulators, thereby enhancing therapeutic efficacy and minimizing side effects, particularly in complex comorbidities.

## Conclusions

In this study, TNFAIP3 was identified as a common key gene of PD and IA by bioinformatics and machine learning methods. Meanwhile, the possible functional roles and immune mechanisms of TNFAIP3 in PD and IA were explored, and promising drugs for the combined treatment of the two diseases were further identified. Our work lays a theoretical foundation for further elucidation of the pathophysiologic mechanisms of PD and IA, and provides new strategies for the diagnosis and treatment of these two diseases.

## Supporting information

S1 Table Five drug molecules predicted based on TNFAIP3. (CSV)

S2 Table Optimal conformations of five drug molecules for targeted binding with TNFAIP3. (CSV)
